# Functional and structural characterization of Hyp730, a highly conserved and dormancy‐specific hypothetical membrane protein

**DOI:** 10.1002/mbo3.1154

**Published:** 2021-02-03

**Authors:** Stewart Fannin, Jonathan Rangel, Abiodun P. Bodurin, Tannon Yu, Brandon Mistretta, Sujina Mali, Preethi Gunaratne, Steven J. Bark, Jerry O. Ebalunode, Arshad Khan, William R. Widger, Mehmet Sen

**Affiliations:** ^1^ Department of Biology and Biochemistry University of Houston Houston TX USA; ^2^ Department of Mathematics University of Houston Houston TX USA; ^3^ Hewlett Packard Enterprise Data Science Institute University of Houston Houston TX USA; ^4^ Department of Pathology & Genomic Medicine Center for Infectious Disease Houston Methodist Research Institute Houston TX USA; ^5^ Present address: Division of Operational Insight Texas Workforce Commission Austin TX USA

**Keywords:** bacteria, dormancy, *Micrococcus luteus*, transmembrane‐spanning helices

## Abstract

Membrane proteins represent major drug targets, and the ability to determine their functions, structures, and conformational changes will significantly advance mechanistic approaches to both biotechnology and bioremediation, as well as the fight against pathogenic bacteria. A pertinent example is *Mycobacterium tuberculosis* (H37Rv), which contains ~4000 protein‐coding genes, with almost a thousand having been categorized as ‘membrane protein’, and a few of which (~1%) have been functionally characterized and structurally modeled. However, the functions and structures of most membrane proteins that are sparsely, or only transiently, expressed, but essential in small phenotypic subpopulations or under stress conditions such as persistence or dormancy, remain unknown. Our deep quantitative proteomics profiles revealed that the hypothetical membrane protein 730 (Hyp730) WP_010079730 (protein ID Mlut_RS11895) from *M. luteus* is upregulated in dormancy despite a ~5‐fold reduction in overall protein diversity. Its H37Rv paralog, Rv1234, showed a similar proteomic signature, but the function of Hyp730‐like proteins has never been characterized. Here, we present an extensive proteomic and transcriptomic analysis of Hyp730 and have also characterized its *in vitro* recombinant expression, purification, refolding, and essentiality as well as its tertiary fold. Our biophysical studies, circular dichroism, and tryptophan fluorescence are in immediate agreement with in‐depth in silico 3D‐structure prediction, suggesting that Hyp730 is a double‐pass membrane‐spanning protein. Ablation of Hyp730‐expression did not alter *M*.* luteus* growth, indicating that Hyp730 is not essential. Structural homology comparisons showed that Hyp730 is highly conserved and non‐redundant in G+C rich Actinobacteria and might be involved, under stress conditions, in an energy‐saving role in respiration during dormancy.

## Graphical abstract

Dormancy requires a bacterium to be tolerant to external stresses, including antibiotics, hypoxia, nutrient deprivation, or immune surveillance from the host organism. Dormant bacteria aim to survive by minimizing their biological events, such as by adopting a state of non‐replicative persistence, yet the molecular mechanism and protein which regulates dormancy are largely unknown. Here, we identified in *Micrococcus luteus* a dormancy‐specific protein, Hyp730 is a membrane protein, widely conserved across Actinobacteria including *Mycobacterium tuberculosis*, but whose biological function has remained elusive. We determined its structural properties which suggest Hyp730‐like proteins are involved in efficient respiration events. Strategies to suppress the function of Hyp730 may represent new therapeutic approaches for inhibiting virulence against pathogens that persist under stress conditions.

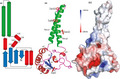

## 1 INTRODUCTION

Bacterial dormancy is a stochastic stress response to counteract harsh environmental conditions including extreme temperature, oxidative stress, lack of nutrients, and antibiotics. Persistent and viable but nonculturable (VBNC) states of dormancy stretch along a continuum, with specific roles as a ‘bet‐hedging’ strategy, whereby a bacterium suffers reduced fitness in typical conditions in exchange for enhanced fitness under stress(es). While many pathogenic and non‐pathogenic bacterial species use dormancy mechanics for survival—including *Staphylococcus aureus*, *Pseudomonas aeruginosa*, *Escherichia coli*, *Micrococcus luteus*, and *Mycobacterium tuberculosis*—the molecular mechanism by which bacteria enter or exit the dormant state is highly elusive. Indeed, the emergence of genes conferring antibiotic resistance in most human pathogens has resulted from dormancy. Despite the extensive ongoing work around the molecular mechanisms of dormancy and antibiotic tolerance, drug targets for unlinking bacterial growth with access to and/or egress from dormancy have been highly rare and unexplored.

Membrane proteins are proven drug targets for persistent infections or dormant bacteria in general. The membrane itself is home to about one‐third of the proteins in the proteome and is the site for critical events: transportation of nutrients and wastes, respiration, generating proton motive force, ATP synthesis, and cell–cell communication in biofilms (Hurdle et al., 2011; Moraes et al., 2014). However, studying membrane protein function is challenging both because of difficulties with functional expression and purification in the quantities needed for biochemical and structural studies (Moraes et al., 2014), and because detergent‐based purification usually strips away native ligands or co‐factors. Research bottlenecks for membrane proteins include limited to no expression, poor extraction success, low purification yields, and paucity of well‐ordered 3D crystals. Furthermore, the microbiology of recalcitrant, dormant pathogen populations poses additional technical roadblocks such as a slow doubling time (over 24 h for *M. tuberculosis*), associated pathogenicity (necessitating a BSL‐3 environment), and poorly defined dormancy states. *M. luteus* NCTC 2665 (MI‐2665) and *Vibrio vulnificus* are the only experimental dormancy models, widely accepted in the field (Ayrapetyan et al., 2015; Mali et al., 2017).

In our earlier work, mapping *M. luteus* proteome changes into and out of dormancy have yielded a proteomic signature: ~20 proteins are upregulated in the nutrient‐deprived dormant state (Havis, Bodunrin, et al., 2019; Mali et al., 2017). In functional characterization studies, proteins we tested appear to be regulatory in events central to dormancy. For example, our knock‐out studies of UspA616 (Havis, Bodunrin, et al., 2019; Mali et al., 2017) and UspA712—both members of a functionally uncharacterized protein family—resulted in severe fitness loss upon starvation and delay of exponential growth in rich media, respectively. All proteins except hypothetical membrane protein 730 (Hyp730) appear to be part of the global shutdown of metabolic, nucleic acid, or protein expression machinery that might have pivotal roles in maintaining long‐term survival during dormancy. Currently, the functional roles of Hyp730 are unknown. The upregulation of its homolog, Rv1234, in *M. tuberculosis* dormancy induced by nutrient starvation had been detected but overlooked in the two decades since its detection (Betts et al., 2002). Hyp730 is highly conserved in high G+C content Actinobacteria with 38% sequence identity, carrying characteristics of two transmembrane‐spanning regions.

Disrupting and disorganizing the structure, function, and location of membrane proteins is a strategy for treating persistent infections and for developing biotechnology applications such as environmental pollution remediation, biofuels, and synthesis of unique molecules. For example, antimicrobials designed to target membrane proteins are promising approaches for treating slow‐growing or dormant bacterial infections and removing industrial/environmental pollution (Hurdle et al., 2011). The clinical effectiveness of this strategy has been demonstrated (Hurdle et al., 2011; Nolan & Walsh, 2009): TMC207 (trade name Sirturo) is an approved medication that disrupts ATP synthesis in *M. tuberculosis* by inactivating ATP synthase (Diacon et al., 2009), and *S. aureus* infections are treated effectively with membrane potential disrupting agents, daptomycin, and telavancin (Steed et al., 2012). Even though most of these membrane proteins from both pathologic and beneficial bacteria are hypothetical—with some yet to be experimentally demonstrated as membrane proteins themselves—the membrane proteome plays a key role in shaping the evolution, variability, and adaptability of slow‐growing or dormant bacteria and in the pathological infections associated with these organisms. Hence, the functional characterization of the ‘hypothetical’ portion of the membrane proteome is a fundamentally important challenge for defining pathogenesis at the molecular level.

Our study describes the protein and mRNA expression profiles of Hyp730, as well as its in silico‐determined 3D‐structure, the latter of which was further validated by biophysical studies. Hyp730 appears to be a transmembrane protein that is uniquely expressed during dormancy and shows no essentiality under non‐stressing growth conditions. Its overall topology is similar to the ActD domain with an unknown role in alternative respiratory complexes which are diverged to save energy during proton transport events—suggesting a potential role in the novel assembly and architecture of these subcellular compartments for efficient energy production during dormancy.

## 2 MATERIALS AND METHODS

### 2.1 Amino acid sequence retrieval, strains, vectors, and chemicals

The translated sequence of Hyp730 and Rv1279 proteins derived from the genome of *M*.* luteus* and *M*.* tuberculosis*‐H37Rv were retrieved from the NCBI GenBank database [GenBank: NC_012803.1 and NC_000962.3 respectively].

Acetate minimal medium (AMM): 4 g/L NH_4_Cl, 1.4 g/L K_2_HPO_4_ at pH 7.4, 0.1 M sodium acetate trihydrate, 0.5 g inosine, 0.5 g yeast extract (0.1%), and 5 ml 100× trace metal stock solution (14.3 g/L MgSO_4_ 7H_2_O, 0.00375 g/L CuSO_4_ 5H_2_O, 0.079 g/L MnCl_2_ 4H_2_O, 0.183 g/L FeSO_4_ 7H_2_O, 0.025 g/L Na_2_MoO_4_, and 0.005 g/L ZnSO_4_ 7H_2_O), was autoclaved, and 0.5 ml filter‐sterilized 1000× vitamin supplement consisting of 0.2 g/10 ml methionine, 0.4 g/10 ml thiamine, and 0.05 g/10 ml biotin dissolved in 0.1 M NaPO_4_ at pH 7.4 was added.

### 2.2 Exponential and dormant states of *M. luteus* MI‐2665 on AMM

A single *M*.* luteus* colony was used to inoculate 5 ml of rich medium broth starter culture. After incubation with vigorous shaking (250 rpm) at 30°C for ~5 h (optical density at 600 nm [OD_600_] of between 0.3 and 0.6), 1 ml of the starter culture was used to inoculate 500 ml of acetate minimal medium (AMM). The 500 ml culture was subsequently incubated at 30°C with rigorous shaking. The bacterial growth was monitored by OD_600_ until the growth curve entered the late stationary phase. When the bacterial growth was in the logarithmic (log) phase (OD_600_ 0.5–0.9), 50 ml of culture was harvested and collected by centrifugation at 10,000 × *g* for 10 min at 4°C. The cell pellet was resuspended in 1 ml of Tris‐EDTA (TE) buffer (100 mM Tris‐HCl pH 8.0, and 10 mM EDTA) and stored at −80°C. *M*.* luteus* cultures were incubated at 30°C, shaking at 250 rpm, for 2 months and then kept at room temperature without agitation for 1 additional month. The VBNC state of *M*.* luteus* was confirmed by plating experiments on rich medium agar plates.

### 2.3 Liquid chromatography‐tandem mass spectrometry

Cell lysate preparation was the same as described in Mali et al. (2017). Briefly, *M*.* luteus* was lysed using a high‐pressure homogenizer, Avestin EmulsiFlex C3, at 30,000 psi. After SDS gel electrophoresis (Mali et al., 2017), the gel‐extracted proteins were subjected to LC‐MS/MS on a Thermo Finnigan LTQ (linear trap quadrupole) linear ion trap mass spectrometer. This system was coupled to an Agilent 1290 Infinity ultraperformance liquid chromatography (UPLC) system using mobile‐phase A and B, 0.1% formic acid in the water, and 0.1% formic acid in methanol, respectively. The raw mass spectrometry data files were transformed to MGF format using the MSConvert tool from ProteoWizard (http://proteowizard.sourceforge.net/tools.shtml). MGF data files were searched using OMSSA against the *M*.* luteus* database containing 2196 proteins (NCBI RefSeq GCF_000023205.1 ASM2320v1) with a randomized decoy database (Geer et al., 2004). The *E* value threshold was adjusted to a false discovery rate (FDR) of <1%. Quantitative‐analysis‐normalized spectral abundance factor (NSAF) (Zybailov et al., 2006) values were natural log‐transformed. NSAF calculations are based on the number of statistically significant spectral identifications annotated to a particular protein divided by the number of statistically significant spectral identifications for all proteins identified in the same sample. There is a normalization factor which is the molecular weight for each protein identified. The number of significant figures presented in Table 1 is used to prevent rounding errors that occur when comparing the small number of spectral identifications of a protein to hundreds and thousands of spectra reliably identified in the actual sample. Such rounding errors potentially obscure real observations of a protein that can be reliably identified and quantitated in the sample. The Shapiro–Wilk test was performed to confirm normal data distribution (http://scistatcalc.blogspot.com/2013/10/shapiro‐wilk‐test‐calculator.html). Proteins that were significantly upregulated were determined by statistical analysis (*p*‐value < 0.05) in all three biological replicates in the dormant or log phases.

**TABLE 1:** Quantitative proteomics comparison of Hyp730 in major growth phases of *M*.* luteus*

Protein description	MW (kDa)	SpC (Dor)	SpC/MW	NSAF (Dor)	NSAF (Stat)	NSAF (Log)	Dormant/Stationary	Dormant/Exponential	Stationary/Exponential
WP_010079730.1 Hyp730 [*Micrococcus luteus*]	18.6	15	0.806451613	0.058522203	0.008742769	0.005258002	6.693783514	11.13012186	1.662754978

Note

The expression level of Hyp730 in three technical replicates of *M*.* luteus* in exponential (Exp), stationary (Stat), and dormant (Dor) growth phases were quantified by normalized spectral abundance factors (NSAF). Relative Hyp730 expression levels in all three‐growth phases are noted.

### 2.4 Phylogenetic tree, hydropathy, and secondary structure analysis

**FIGURE 1:**
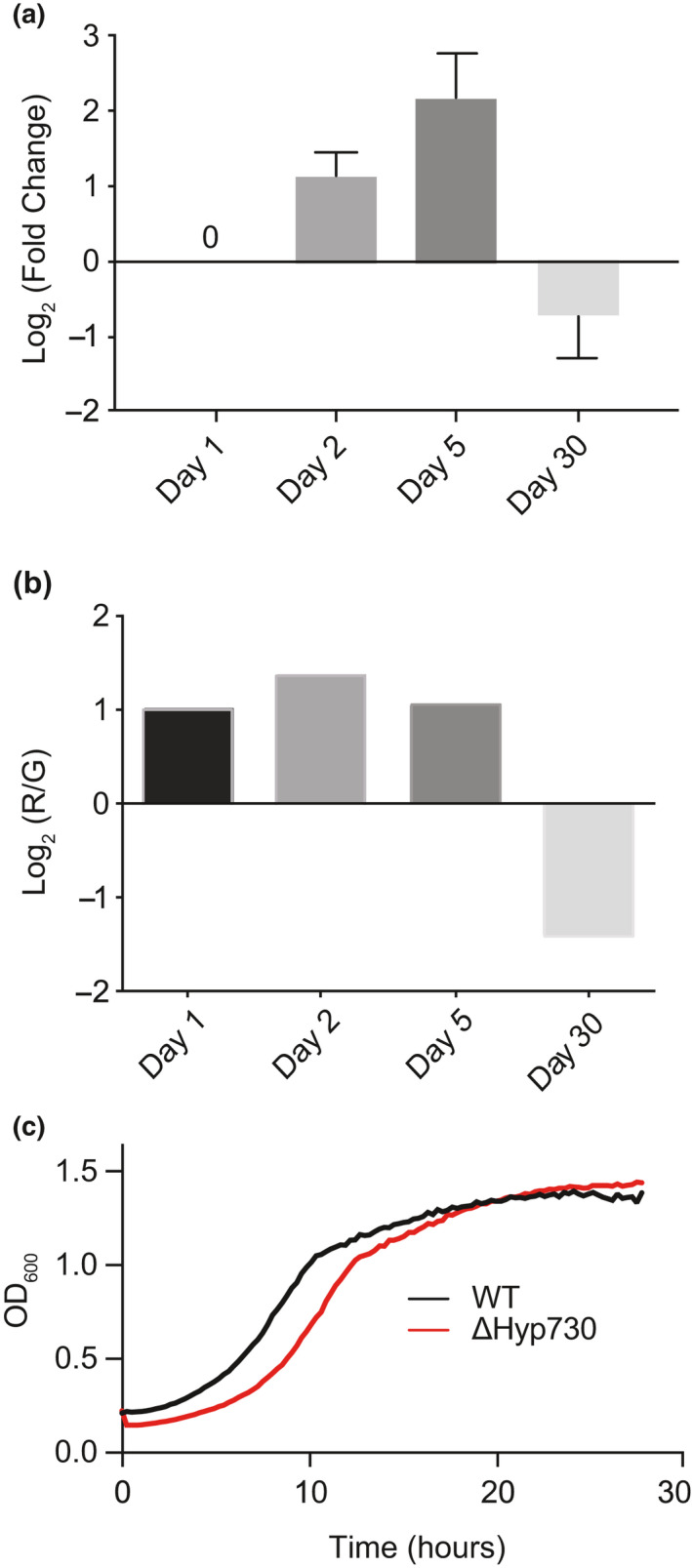
Differential Hyp730 RNA expression level probed in different growth phases of *M*.* luteus* by (a) qRT‐PCR amplification and (b) RNA‐sequencing. In both tests, changes in expression levels are shown in log‐scale. (c) Growth curve comparison of WT and ∆Hyp730:kan strains in rich LB media

The NCBI Blastp tool was used to identify the sequence homologs of Hyp730 (PMID: 9254694), and the BLAST tree view was used to construct a circular phylogenetic tree that uses distance divergences. ClustalX (Larkin et al., 2007) was used to perform the multiple alignments with proteins related to *M*.* luteus* Hyp730 and to construct the alignment in Figure 1. Bootstrapping by the neighbor‐joining algorithm was used to produce an output file. Ancestor sequence was calculated using secondary structure propensity and the hydropathy plot was constructed using AL2D and Aln2plot for the aligned Hyp730 protein family (Zimmermann et al., 2018).

### 2.5 RNA‐sequencing library preparation and sequencing

Extracted RNA samples underwent quality control assessment using the RNA tape on a Tapestation 4200 (Agilent) and were quantified with a Qubit Fluorometer (Thermo Fisher). The RNA libraries were prepared and sequenced at the University of Houston Seq‐N‐Edit Core per standard protocols. RNA libraries were prepared with QIAseq Stranded Total RNA Library Kit (Qiagen) using 100 ng of input RNA. The size selection for libraries was performed using SPRIselect beads (Beckman Coulter), and the purity of the libraries was analyzed using the high sensitivity DNA 1000 tape on the Tapestation 4200 (Agilent). The prepared libraries were pooled and sequenced using NextSeq 500 (Illumina), generating ~12 million 2 × 76 bp paired‐end reads per sample.

### 2.6 RNA‐Sequencing transcriptome analysis

The RNA‐seq raw fastq data were processed with CLC Genomics Workbench 12 (Qiagen). The Illumina sequencing adaptors were trimmed, and reads were mapped to the NCTC 2665 *Micrococcus luteus* reference genome. Read alignment was represented as integer counts by using parameters of mismatch cost 2, insertion cost 3, deletion cost 3, length fraction 0.8, similarity fraction 0.8, and a maximum of 10 hits for a read. Reads with <10 hits were removed. Integer read counts were normalized by the Trimmed Means of M‐values (TMM) algorithm where a trimmed mean (M) is the average expression of a gene after 30% of the upper and lower data is removed (Robinson & Oshlack, 2010). Once trimmed, a scaling factor was calculated based on the weighted mean, with weight estimated by the asymptotic variance of log ratios between the reference samples, exponential phase, and the test samples, stationary phase at 36 h and 5 days, and dormancy at 30 days. After normalization, we performed differential gene expression using the EdgeR package which uses a generalized linear model linked to the negative binomial distribution to identify significance by testing for differential expression using the exact test (Robinson et al., 2010). The significance level of FDR adjusted *p*‐value of 0.05 and a log_2_‐fold change >2 was used to identify differentially expressed genes.

### 2.7 RNA Extraction and quantitative real‐time PCR

Total RNA was extracted from *Micrococcus luteus* NCTC 2665 (*M*.* luteus*) grown in minimal media acetate and LB media at different stages of growth: exponential, stationary, 5 days stationary, and dormancy phase. Cells were pre‐treated with 1% lysozyme and incubated for 10 min at 37°C. One milliliter Trizol reagent (Invitrogen) was added to resuspend the cells and homogenized in the tissue lyser at a frequency of 30 hertz for 5 min. The homogenized mixture was left at room temperature for 5 min to allow complete dissociation of the nucleoprotein complex. Subsequently, 0.35 ml of chloroform was added, mixed, and allowed to sit at RT for another 3–5 min. The chloroform:isoamyl alcohol (24:1) steps were repeated three times. Finally, the RNA pellet was dissolved in 40 μl RNase‐free water. This was immediately followed with on‐column RNase‐Free DNase I (Qiagen) digestion. qRT‐PCR was used to confirm the differential gene expression of the Hyp730 gene from previously stated sample cultures. cDNA synthesis was performed using a high capacity cDNA reverse transcription kit (#4368814, Applied Biosystems). cDNA was subjected to qRT‐PCR using Brilliant III Ultra‐Fast SYBR Green qPCR Master Mix (#600892, Agilent Technologies) in a StepOnePlus Real‐Time PCR System (v. 2.0, Applied Biosystems). Normally, all real‐time PCR reactions were carried out as 10 μl reactions in 96‐well plates. Each reaction mixture contained 1 μl diluted cDNA, 2 μl each of forward and reverse primers (10 μM), 7.5 μl 2×SYBR Green PCR Master Mix, and 2.5 μl water. Normalization was performed using chromosomal DnaA mRNA levels.

#### 2.7.1 Cloning of Hyp730

The Hyp730 open‐reading frame was amplified from the purified *M*.* luteus* genome using forward and reverse primers, 5′‐AAAGGAGTACTCCCATGGCCAGCC‐3′ and 5′‐GGGCCCTTCTTCGGTGGACTTTCGTG‐3′ via PFU polymerase. This amplified DNA was then cloned into the pET‐14b vector with NcoI and EcoRV (Promega #R635A) using T4 DNA ligase (NEB #M0202S). Due to poor, insoluble, and heterologous expression, Hyp730 was subcloned into an *E*.* coli*‐based membrane protein overexpression system that carries an engineered bacterial outer membrane protein F (pOmpF) (Su et al., 2013). PCR amplification of Hyp730 with the forward and reverse primers 5′‐caagcatatgATGgccagccccgaggcc‐3′ and 5′‐gattggatcctcagtctccggggtgggc‐3′ were used to generate a NheI/BamHI cleavable fragment, which attaches to the pOmpF tag, and a linker sequence [GGGGGLVPRGSGTTSASGS] containing a polyglycine linker and thrombin cleavage site. The integrity and completeness of the clones were verified by sequencing.

### 2.8 Overexpression and purification of Hyp730

The clone of pOmpF‐Hyp730 fusion was transformed into *E*.* coli* Rosetta 2 (Novagen) host cells compatible with *lac* operon expression systems. A 5 ml starter culture of LB media containing 50 μg/ml kanamycin was inoculated using a single colony for overnight growth at 37°C. The starter culture was subsequently used the following day to inoculate a larger LB culture at 1/1000 dilution, which was grown to OD_600_ 0.5–0.6 at 250 rpm and 37°C before adding 1 mM IPTG to turn on the *lac* operon. The IPTG‐induced cells were harvested, resuspended in Buffer A (50 mM Tris, pH 8.0, 150 mM NaCl), and lysed using the Avestin EmulsiFlex C3 at 10,000 psi. The cell lysate was centrifuged at 20,000 × *g* for 20 min at 4°C to pellet inclusion bodies. To determine the best conditions for solubilizing the Hyp730 membrane protein, the pellet was resuspended in 50 mM Tris buffer and 1 ml of the resuspended cell debris was transferred to microcentrifuge tubes. Samples were then spun down and the resulting pellet was resuspended in 250 μl of the following six test detergents: LDAO (N,N‐dimethyl‐dodecylamine), Anzergent (N‐Dodecyl‐N,N‐dimethyl‐3‐ammonio‐1‐propanesulfonate), Zwittergent (N‐tetradecyl‐N,N‐dimethyl‐3‐ammonio‐1‐propanesulfonate), Tween 20, Triton‐X, and SDS, with the seventh sample being resuspended in 6 M urea as a control. Samples were allowed to solubilize while shaking at room temperature. Aliquots of the soluble fraction were collected at the following time points: 1 h, 4 h, and overnight. These aliquots were then run on SDS‐PAGE to check for solubilization of the Hyp730 protein.

The pellet was washed multiple times with Buffer A and MilliQ water and dissolved in Tris‐buffered saline with 6 M urea and 0.1% LDAO (Figure A2a). The solubilized protein was refolded by removing the urea via dialysis into Tris‐buffered saline, and the pOmpF tag was removed by overnight digestion with thrombin (Figure A2b). Precipitate resulting from the thrombin digestion was resuspended in Tris‐buffered saline with 6 M urea and 0.1% LDAO, and the refolding process was repeated. Finally, Hyp730 was isolated from pOmpF using Mono‐Q anion‐exchange chromatography (Figure 3). For each Hyp730 purification, SDS‐PAGE bands or FPLC fractions that corresponded to the purified proteins were trypsin digested. The digested peptides were separated in reverse‐HPLC and then peptide sequences and any contaminants were identified using MS/MS spectra. In Figure A2d, peptides corresponding to the F34–R46 range identified the protein from the excised band in the SDS‐PAGE.

### 2.9 Circular dichroism measurements

The circular dichroism measurements were carried out using an RSM1000 spectrometer, which was calibrated with ammonium (+)‐10‐camphorsulfonate. 9.6 μM Hyp730 in a 1 mm cuvette was used to collect the CD spectra wavelength ranging from 200 to 240 nm and then normalized against the buffer spectra. Each spectrum was an average of three scans to minimize errors. Per residue molar ellipticity was calculated from the observed ellipticities according to the following equation:

$$\left[q\right]\;=\;qo\;\times \;Mr/lc$$

[*q*] is the per residue molar ellipticity in deg.cm^2^.mol^−1^, *qo* is observed ellipticity in mdeg, *Mr* is the molecular weight of Hyp730, *l* is the path length in centimeters, and *c* is Hyp730 concentration in mol/L. The percentage of secondary structure was calculated using the CD Pro analysis suite reference set SMP50 to compare the spectra of Hyp730 to that of proteins with a known structure including other membrane proteins (Sreerama & Woody, 2000, 2004). Voltage changes in the CD detector or oscillations during scans were constantly monitored to eliminate any detector saturation and ensure that sufficient light reached the detector during CD data acquisition.

### 2.10 Tryptophan fluorescence

Fluorescence spectra were recorded with the RSM1000 spectrometer in a quartz cell with a 1 cm path length. After incubating Hyp730 at room temperature for 15 min, the spectra were recorded for tryptophan fluorescence measurements at excitation wavelength of 295 nm and an emission range from 285 to 435 nm. The protein concentration used was 10 μM, and the measurement was carried out at 25°C.

### 2.11 Protein structure prediction

The protein structure predictions were performed using web interfaces of the programs available in public domains. The structure prediction programs used were: Robetta (Kim et al., 2004), I‐TASSER (Iterative Threading ASSEmbly Refinement) (Yang et al., 2015), and Phyre version 2 (Kelley et al., 2015). The electrostatic surface (−5 [red] to +5 [blue] kT/e) was calculated using the Adaptive Poisson–Boltzmann Solver (APBS) software plug‐in for PyMOL (Baker et al., 2001).

### 2.12 Whole‐genome sequencing

Extracted genomic DNA (gDNA) was quantified by the Qubit 2.0 fluorometer (Thermo Fisher), and integrity was verified using a 4200 TapeStation (Agilent) genomic DNA assay. Sequencing libraries were prepared using the Qiaseq FX DNA library kit (Qiagen) per standard protocol. One microgram of gDNA was fragmented, Illumina adapter‐ligated, and amplified. The size selection for libraries was performed using SPRIselect beads (Beckman Coulter), and the purity of the libraries was analyzed using the high sensitivity DNA 1000 tape on the Tapestation 4200 (Agilent). Libraries were sequenced on a MiSeq (Illumina) and assembled using the SPAdes genome assembly algorithm.

## 3 RESULTS

### 3.1 Proteomics analysis of Hyp730

Our quantitative proteomic analysis measures the differential quantities of expressed proteins in various *M*.* luteus* growth phases. A 4‐fold decrease of protein diversity was observed in the MI‐2665 VBNC dormancy state compared to the exponential and stationary growth phases. The detected number of proteins in both logarithmic and stationary growth phases was about 700, while ~200 were seen in dormant cells (Mali et al., 2017). Hyp730 is the only membrane protein upregulated in the dormant phase with its expression 7‐ and 11‐fold higher in the dormant phase in comparison to the stationary and exponential phases, respectively, with a statistical *p*‐value of <0.05 (Table 1).

### 3.2 Differential gene expression of Hyp730 in the different *M. luteus* growth stages

In a sensitive and comprehensive approach to analyzing the dormancy adaptation of *M*.* luteus*, we monitored the initial transcriptional response to minimal media. The differential gene expression patterns of Hyp730 in each growth stage of the exponential (day 1), stationary (day 2), late stationary (day 5), and dormancy (day 30) phases in minimal media acetate were examined by whole transcriptome sequencing (RNA‐seq) and quantitative PCR (qRT‐PCR) studies. These time points were chosen to closely follow gene expression alterations as well as to ensure abundant RNA isolation at good quality. The time points selected here are in parallel to the dormancy model in Mali et al. (2017), where the overall proteome content of *M*.* luteus* lessened rapidly over the first 96 h.

**FIGURE A1:**
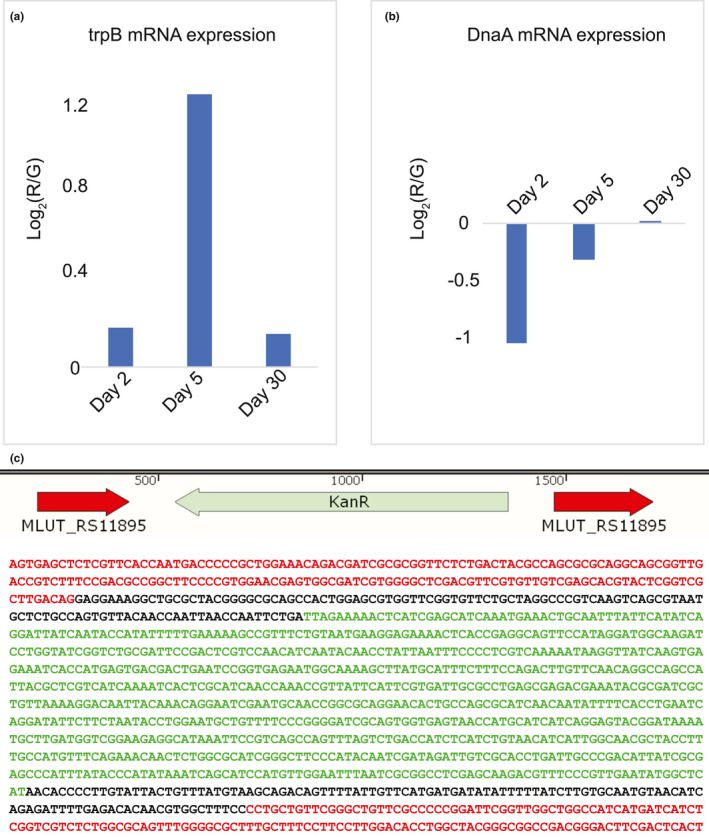
mRNA transcript level of housekeeping genes and generation of ∆Hyp730 *M*.* luteus*. Housekeeping gene (a, trpB and b, DnaA) levels did not change significantly in the stationary, late stationary, and dormant growth phases relative to the exponential stage. (c) Insertion of kanamycin cartridge while the generation of ∆Hyp730:kan strain was confirmed using whole‐genome sequencing

In comparison to the exponential growth phase, the Hyp730 RNA transcript level increased by 2‐fold and 5‐fold in the stationary and late stationary phases, respectively (Figure 1a). These changes are highly similar to the observed RNA transcript level change of the Hyp730 homolog in *M*.* tuberculosis*, Rv1234 (Betts et al., 2002). In the dormant phase, Hyp730 RNA leveled back to that of the baseline expression of the transcript (Figure 1a). The expression of the Hyp730 gene is consistent with the RNA‐seq data displaying upregulation at day‐2 and day‐5, but transcript expression is downregulated as the cells enter day‐30 (Figure 1b). Housekeeping gene levels did not change more than 1.5‐fold, as expected (Figure A1a,b).

### 3.3 Generation of ∆Hyp730 *M. luteus* and assessing its essentiality in growth

The Hyp730 gene (Mlut_RS11895) was modified by insertion of a kanamycin resistance cartridge via homologous recombination as in our previous study (Havis, Bodunrin, et al., 2019; Havis, Rangel, et al., 2019). Colonies that grew under kanamycin selection were subjected to whole‐genome sequencing to confirm the Hyp730 knock‐out strain of *M*.* luteus* (∆Hyp730). Sample #2 has a confirmed sequence on contig NODE 30 containing the kanamycin resistance gene wrapped by the Hyp730 (MLUT_RS11895) gene sequence on either side as expected (Figure A1c).

The phenotype of the ∆Hyp730 *M*.* luteus* strain was tested in lysogeny broth, which showed no alteration or growth defect in comparison to the wild‐type (WT) *M*.* luteus* (Figure 1c).

### 3.4 Expression and purification of the recombinant pOmpF‐fusion Hyp730

**FIGURE 2:**
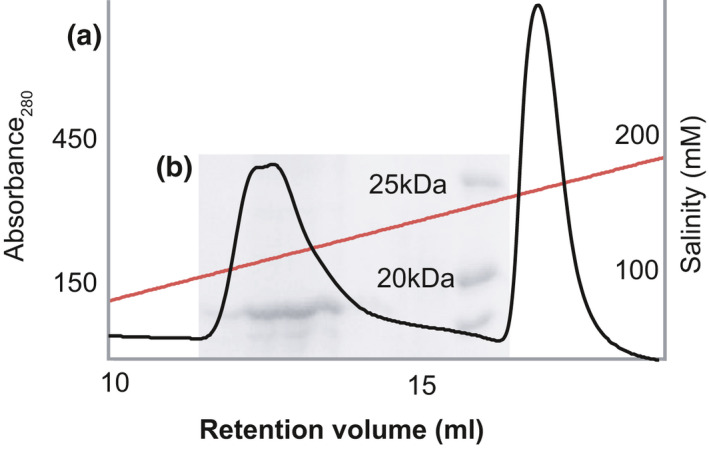
Purification of Hyp730. (a) Ion‐exchange purification of Hyp730. The first peak eluted with salt is Hyp730 at 20°C. (b) SDS‐PAGE of the Hyp730 fractions showing more than 95% purity

**FIGURE A2:**
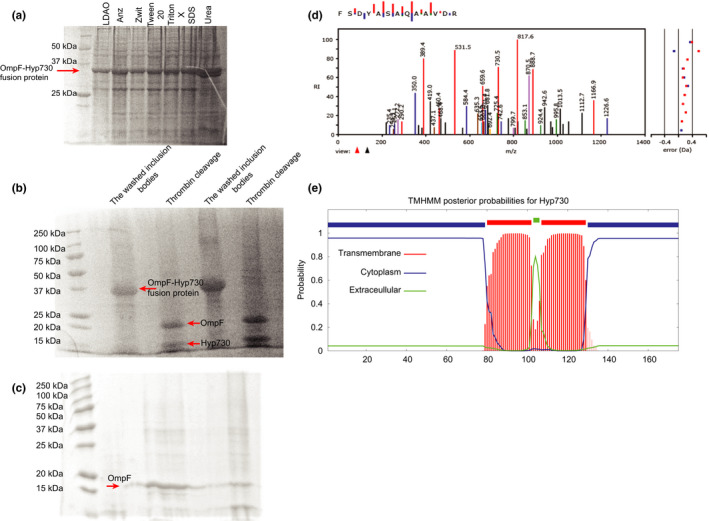
The extent of detergent‐solubilization, thrombin cleavage, absolute identification of the purified Hyp730, and prediction of Hyp730 membrane topology with Hidden Markov modeling. (a) Solubilization of Hyp730 was probed in LDAO, Anzergent, Zwittergent, Tween 20, Triton‐X, SDS, urea. (b) Thrombin cleavage reaction of the refolded OmpF‐Hyp730, (c) the final ion‐exchange purification and (d) identification of the top band in the SDS‐PAGE using mass spectroscopy. (e) The posterior probability for transmembrane helix, cytoplasmic, or extracellular domains displayed for the Hyp730 from *Micrococcus luteus*

In our initial test, the recombinant expression of Hyp730 in *E*. *coli* resulted in a low to non‐existent yield. Since Hyp730 carries two predicted transmembrane helices, we utilized the ~18 kDa N‐terminal fragment of the complete bacterial outer membrane protein F (pOmpF) and generated pOmpf‐fused Hyp730—which contains a polyglycine linker with a thrombin cleavage site—to increase and direct its recombinant expression into inclusion bodies (Su et al., 2013). The expression yield of the pOmpF‐fused Hyp730 fusion was 10 mg/L. Solubilization of the pOmpF‐fused Hyp730 protein was tested in multiple detergents. Although several of these detergents appeared partly promising in the solubilization assay—dodecyldimethylaminoxid (LDAO) being the best among them—most have low stabilizing effects on the fusion proteins (Figure A2a). Therefore, 6 M urea was further supplemented to ensure the complete denaturation and solubilization of the pOmpF‐Hyp730 and subsequently removed slowly by dialysis. The final concentration of LDAO left after urea dialysis was 0.1%, which is 4× the critical micelle concentration (CMC) (0.023%). To cleave off the pOmpF, the cleavage reaction composed of thrombin/pOmpF‐Hyp730 in a 1/100 (w/w) ratio was carried out in TBS containing 25 mM CaCl_2,_ which resulted in precipitation of the pOmpF protein with most of the Hyp730 remaining in‐solution. Based on its ability to maintain a 3D‐fold, stability at 4°C, and compatibility with biophysical experimental methods, LDAO detergent was chosen for further studies. The predicted molecular weight of Hyp730 is 19.2 kDa, which does not correlate with observed molecular weights in SDS‐PAGE (Figure A2b,c)—a phenomenon known as gel‐shifting, a commonly observed behavior of membrane proteins in SDS‐PAGE due to hairpin formation of the transmembrane regions in detergents like SDS (Rath et al., 2009). Nonetheless, the identity of the cleaved Hyp730 band from SDS‐PAGE was confirmed using mass spectroscopy (Figure A2d). In the final step of purification, ion‐exchange (Mono‐Q) chromatography removed residual pOmpF and thrombin using a NaCl gradient (Figure 2a). Isoelectric points of pOmpF and Hyp730 are 5.7 and 5.1, respectively. Hyp730 was eluted as a single peak at 200 mM NaCl (Figure 2a,b).

### 3.5 Secondary structure analysis of Hyp730

**FIGURE 3:**
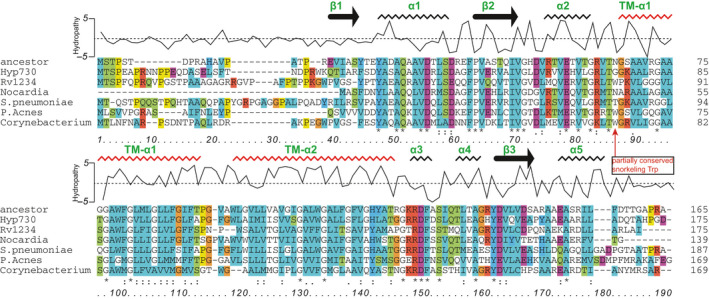
Sequence alignment of Hyp730 with relevant homolog proteins. Averaged hydropathy plot showing increased hydrophobicity around transmembrane. Predicted secondary structures are noted. Similar residues in alignment are colored similarly using ClustalX (Larkin et al., 2007) and noted as ‘:’, identical residues are noted as ‘*’, and sequence homology are shown as bars below the alignment. Partially conserved snorkeling Trp residue is noted by a red arrow

**FIGURE 4:**
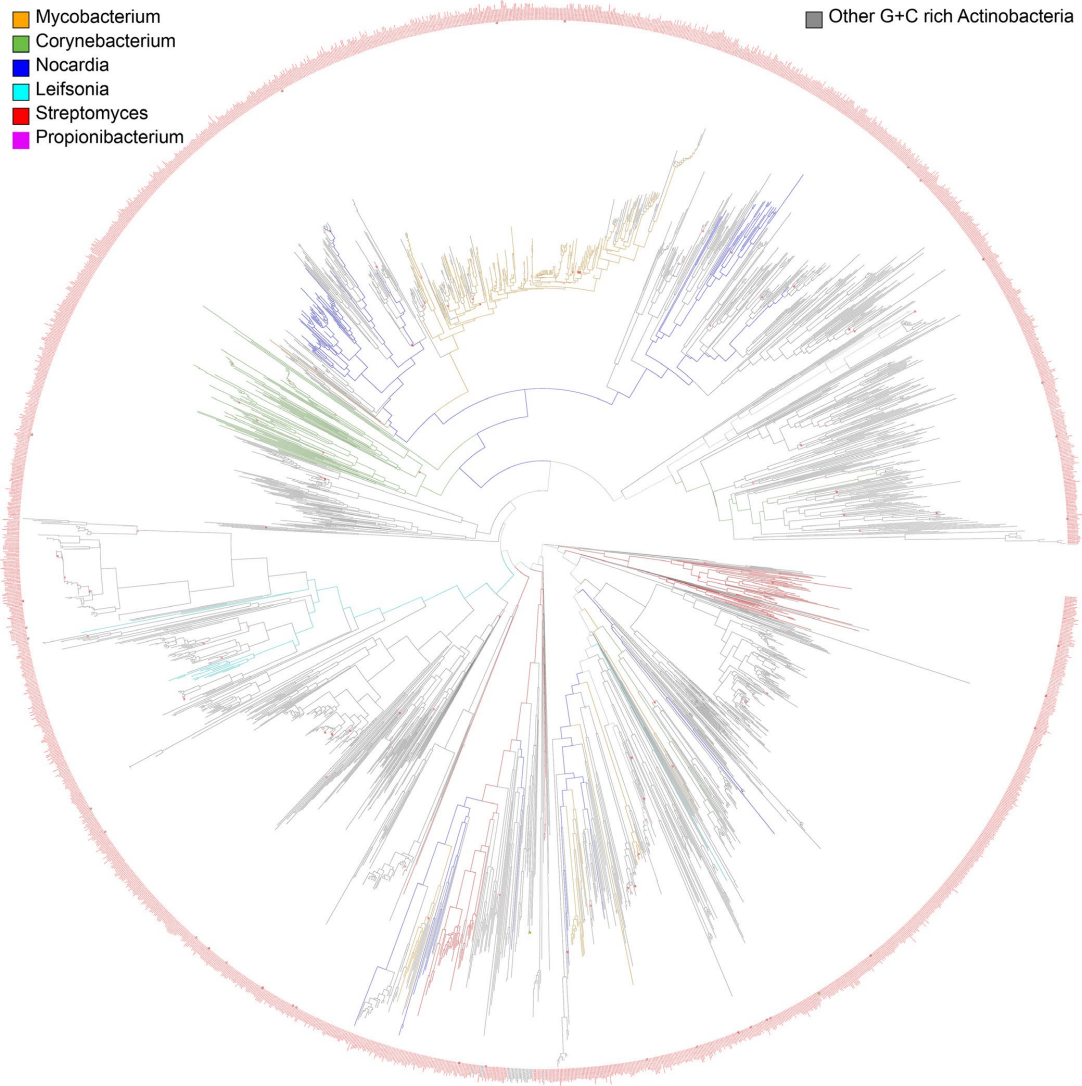
Rooted circular phylogenetic tree of Hyp730 homologs with divergence signified as distance. All bacteria are G+C rich group with the Actinobacteria. Some bacteria genera are colored

The sequence comparison and phylogenetic tree constructed with an *E*‐value threshold of 10^−6^ showed a highly congruous sequence alignment in 3014 Gram‐positive bacteria with high G+C content in their DNA, a group which includes *M*.* tuberculosis*, *P*.* acnes*, *S*.* pneumonia*, and *C*. *bovis* (Figure 3). All of the sequence homologs are categorized as hypothetical proteins from Actinobacteria, a cosmopolitan bacterial phylum and one of the largest among aquatic and terrestrial ecosystems (Figures 3 and 4). Hydropathy, a membrane protein topology prediction method based on hidden Markov modeling (using TMHMM server—http://www.cbs.dtu.dk/services/TMHMM/) (Krogh et al., 2001) and secondary structure topology predictions of Hyp730 and its homologs suggest that the Hyp730‐like protein family contains two transmembrane helices and that their cytoplasmic domain folds into four anti‐parallel β‐strands sandwiched by five amphipathic helices (Figure 3, Figure A2e). An independent 3D‐structure prediction of Hyp730 using the Robetta, I‐TASSER, and Phyre3D servers agree with the secondary structure analysis.

### 3.6 In silico structure Hyp730

**FIGURE 5:**
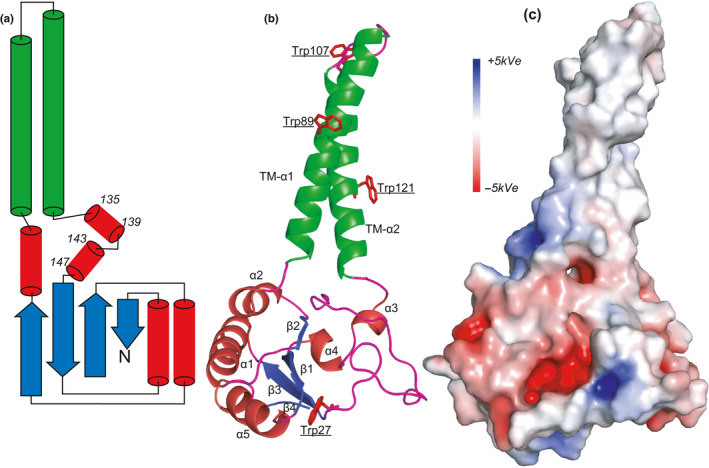
In silico 3D‐structure of Hyp730. (a) Topology diagram of Hyp730 showing α‐helices in red, β‐strands in blue, transmembrane α‐helices in green, and loops in magenta. (b) Secondary structure representation of Hyp730 colored the same as in the topology diagram. Trp residues are shown in red sticks. (c) Electrostatic potentials mapped to the molecular surface of the predicted structure of Hyp730 [positively charged regions are colored in blue, (>+5 kT/e) and negatively charged regions in red (>−5 kT/e)], which were calculated using the Adaptive Poisson–Boltzmann Solver software plug‐in for PyMOL (Baker et al., 2001)

**FIGURE A3:**
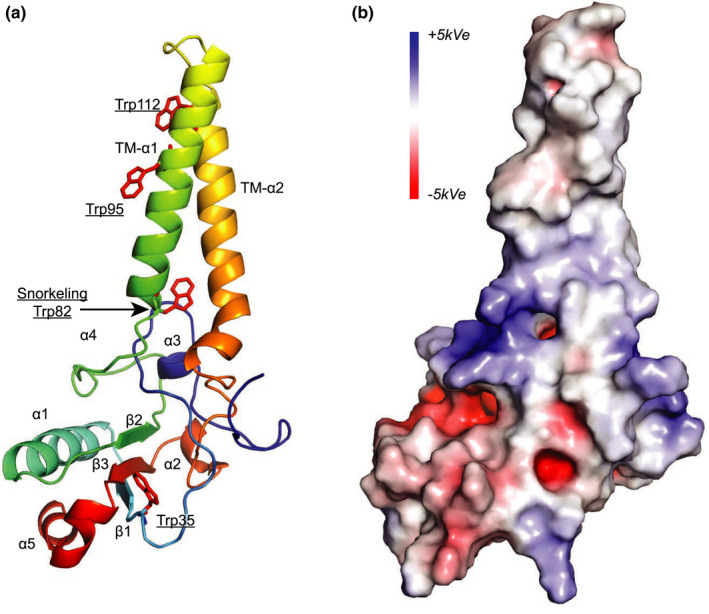
In silico structure of Rv1234. (a) Cartoon representation and (b), the electrostatic surface of Rv1234. Electrostatic surface (−5 [red] to +5 [blue] kT/e) was calculated using the Adaptive Poisson‐Boltzmann Solver software plug‐in for PyMOL (Baker et al., 2001)

**FIGURE A4:**
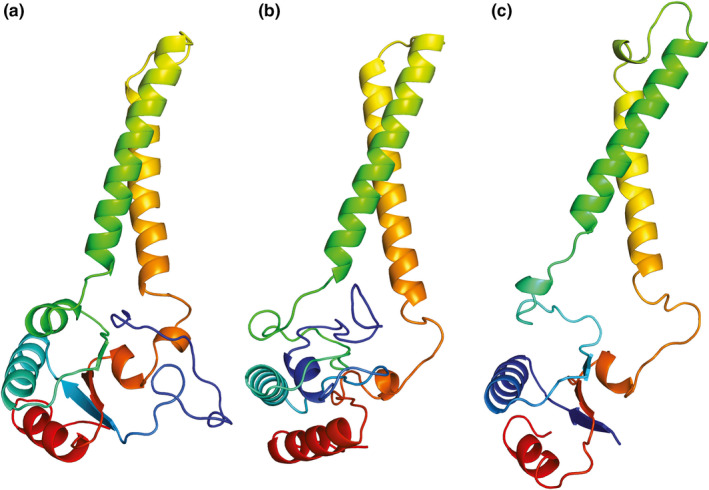
Superimposed structures of Hyp730 modeled by (a), Robetta, (b), Phyre2, and (c), I‐TASSER

A 3D homology search for Hyp730 and Rv1234 using servers such as PDBeFold or Swiss‐model workspace did not identify any significant hit to a template protein having a crystal or NMR structure with acceptable sequence homology. To model the Hyp730 structure, we used three independent approaches that employ unique algorithms to determine in silico structures from protein primary sequences: (i) Robetta, which utilizes a fragment‐insertion strategy to construct protein structures in both template‐based and de novo modes, (ii) Phyre2, which uses homology modeling using HH‐suite for protein sequencing search, and (iii) I‐TASSER, which detects structure templates using fold‐threading from a protein data bank and reassembles the query using replica exchange Monte Carlo simulations (Figures A3 and A4). All three unique methods generated highly similar 3D‐models for both proteins that have one globular domain—composed of three central β‐strands sandwiched by amphipathic helices—and two hydrophobic helices that distinctly protrude, are independent of the globular domain (Figure 5a), and are linked by a six or seven‐residue linker containing a proline residue (Figure 3a‐red annotation). The two linkers connecting the cytoplasmic globular domain to the membrane helices are more than 7 residues in length (Figure 5b). The electrostatic surface potentials mapped to the predicted Hyp730 structure show negatively charged patches between the transmembrane and cytoplasmic regions and large areas of positive potential on the rest of the cytoplasmic domain (Figure 5c).

### 3.7 Probing structural features of Hyp730 by fluorescence and circular dichroism

**FIGURE 6:**
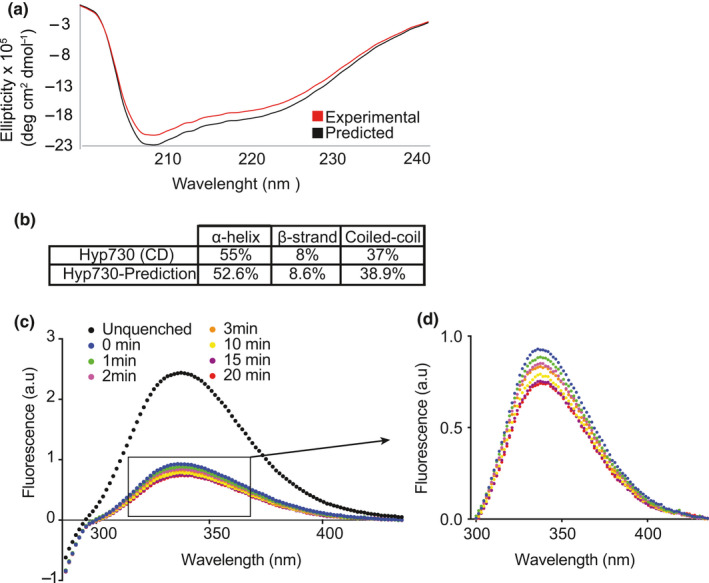
Circular dichroism and fluorescence spectra of Hyp730. (a) CD spectra represent an average of three scans. (b) Theoretical secondary structure content. (c) Trp fluorescence spectrum of wild‐type and reduced Hyp730, (d) inset showing the time‐dependent reduction of Trp fluorescence

**FIGURE A5:**
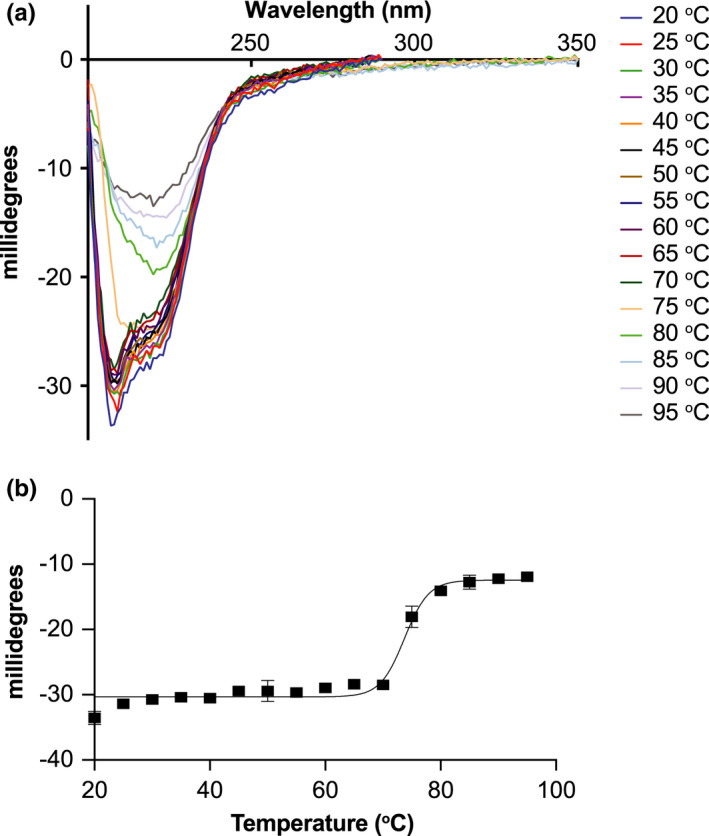
Thermal melt plots of Hyp730. (a) Overlaid CD spectra obtained at different temperatures ranging from 20 to 95°C. (b) Change of the spectral peak at 208 nm plotted as a function of temperature and, and the curve was fit to a sigmoidal function (*R* ^2^ = 0.9725). The calculated Tm is 73.8°C

We characterized the secondary structure content of proteins using far UV circular dichroism (CD) spectra, which provide quantitative estimation by detecting excitation of the backbone amide chromophore. The experimental CD spectrum shows negative bands at 208 and 222 nm for α‐helices and 215 nm for β‐strands (Sen et al., 2009). We utilized the CDpro suite (Sreerama & Woody, 2000, 2004), which provides a reference set of proteins for higher validity and reliability and showed 55% helical and 8% strand content, consistent with the predicted 3D‐structure (Figure 6a,b). Moreover, CD band changes in thermal melt steps were also monitored to further confirm that the tertiary, and not just secondary structure, has been properly formed. The melting temperature of Hyp730 appears to be at 73°C (Figure A5a,b).

As further validation that Hyp730 forms a stable tertiary fold, tryptophan (Trp) emission spectra in the presence of a reducing reagent, dithiothreitol (DTT), at different time points were collected. In the in silico Hyp730 structural model, Trp82 and Trp121 are completely buried, Trp107 is partially so, and Trp27 is solvent accessible (Figure 5b). A tryptophan residue that is freely diffusing (or located in the unfolded polypeptide chain) shows emission maxima at 359 nm when excited at 295 nm, but the emission maxima of Hyp730 showed more than 20 nm blue‐shift to 337.5 nm. This observation strongly suggests that Hyp730 adopts a folded conformation (Figure 6c). Secondly, we used DTT as a static and selective quencher. Since DTT does not penetrate the environment of buried tryptophan residues, only a limited reduction of Trp emission—most probably resulting from the quenching of two solvent‐exposed Trps—was observed after 20 min of DTT treatment (Figure 6d).

## 4 DISCUSSION

Membrane proteins, by their intrinsic hydrophobic nature, exist within lipids and have low solubility in aqueous environments. The family of Hyp730 homologs contains two regions with high helical propensity and hydrophobicity (Figures 1 and 4). Thrombin cleavage of pOmpF‐Hyp730 resulted in precipitation of pOmpF, a complete integral membrane protein with a negative Grand Average of Hydropathy (GRAVY) (Kyte & Doolittle, 1982) value for pOmpF at −0.4. Nevertheless, Hyp730 has a positive GRAVY score of 0.13 and stayed soluble after thrombin digestion despite having two predicted transmembrane helices, suggesting that the two flanking regions of the hydrophobic helices are located in the cytoplasm. Hyp730 was eluted in the final ion‐exchange step (Figure 3a), further suggesting the cytoplasmic globular domain of Hyp730 does not aggregate when exposed to an aqueous solution and helps to maintain its native conformation.

While cytosolic proteins have a low Trp content (~1.2%) (Schiffer et al., 1992), Trp residues on average make up ~5.2% of every known transmembrane helices (Schiffer et al., 1992). The transmembrane helices for Hyp730 and its Mtb homolog, Rv1234, have ~6% Trp content (Figure 1a,b). Additionally, Trp residues in membrane proteins can preferentially reside near the lipid–water interface (de Jesus & Allen, 2013) and are thought to be responsible for anchoring the helices to the membrane. Additionally, the structure models for Rv1234 and other Hyp730 homologs in the Actinobacteria phylum exhibit strong evidence for the partially conserved ‘snorkeling’ Trp residue located near the membrane (Figure 3 and Figure A3a). This residue could interact differently with the transmembrane regions in the kink‐angle between two transmembrane helices and enforce different conformational presentations of the cytoplasmic domain. For example, the membrane thickness is altered in dormancy: triacylglycerols (triglycerides or TAG) are accumulated at a substantial quantity in the Mtb membrane under dormancy (Maurya et al., 2018).

In our proteomic studies of the dormant *M*.* luteus*, our mass spectroscopy analysis repeatedly identified peptides from the cytoplasmic domain of Hyp730; yet, no peptides were detected for the predicted transmembrane helices. For instance, FSDYASAQAAVDRLSDAGFPVER and DFDSLQTLEAGHYEVQVEAPYAAEAAR are the two major peptides detected, which reside in both flanking regions of TM helices (Figure 1). Given the inherent challenges associated with the detection of membranomes (e.g., hydrophobicity, inefficient protease cleavage), detectable peptides in mass spectroscopy are mostly limited to the cytoplasmic sequences (Carroll et al., 2007), suggesting that the two hydrophobic regions reside within the cell membrane.

Although the number of proteins declined substantially in our proteomics profiling, the expression level of Hyp730 increased 7‐ and 11‐fold in the dormant state relative to the stationary and exponential phases, respectively. Betts et al. 2003 reported (Betts et al., 2002) that Rv1234, the Mtb homolog of Hyp730, exhibits increased upregulation by approximately 5‐fold in *M*.* tuberculosis* dormancy‐induced nutrient starvation, similar to Hyp730 upregulation in the dormant stage in minimal acetate media. *M*.* luteus* has high homology (near sequence identity) with the pathogenic species *M*.* tuberculosis*, *M*.* bovis*, and *M*.* leprae*, and more critically shares a similar dormancy mechanism (Figure 1). Particularly, Rv1234 and Hyp730 share 38% identity and 60% sequence homology with an Expect (*E*) value of 3 × 10^−23^. In brief, Rv1234 and Hyp730 are most likely paralogues (not orthologues) and have the same physiological function.

Gene expression analysis showed upregulation of the Hyp730 mRNA level as the *M*.* luteus* culture increased its optical density, and Hyp730 seemed to be transcribed to a greater level before reaching dormancy. In rich media where reaching a complete dormant bacterial ensemble is not possible due to an overabundance of a non‐depleting nutrient source, the Hyp730 mRNA are constantly transcribed. It is certainly plausible to synthesize dormancy‐specific membrane proteins earlier in the bacterial life cycle since (i) protein synthesis, along with many physiological metabolic events, are extensively slowed to a minimum efficiency during dormancy, and (ii) membrane proteins are challenging to express, refold, and translocate to the membrane at a singularly slow metabolic state.

Based on our sequence analysis, Hyp730 homologs are found in pathogens of the phylum Actinobacteria (e.g., Corynebacterium, Mycobacterium, Nocardia, and Propionibacterium) as well as in soil inhabitants (Streptomyces), plant commensals (Leifsonia), and gastro‐intestinal commensals (Bifidobacterium) (Figure 4). Within Actinobacteria, the trifecta of the Corynebacterium, Mycobacterium, and Nocardia genera together form a monophyletic taxon called the CMN group (Ventura et al., 2007). CMN share a unique waxy envelope that makes them causative pathogens of diphtheria, tuberculosis, and nocardiosis (Ventura et al., 2007). Hyp730 is conserved in this high G+C DNA content Gram‐positive phylum (Figure 4). Since the divergence of Actinobacteria from other phyla is quite ancient, and delineating the phylogenetically closest bacterial group to Actinobacteria with confidence is not possible (Ventura et al., 2007), the emergence of Hyp730 within the Actinobacteria appears to be recent.

We utilized multiple 3D‐structure prediction algorithms to model Hyp730 structures. The cytoplasmic domain contains three anti‐parallel strands sandwiched by amphipathic helices and two membrane‐spanning helices protrude from the globular cytoplasmic domain, which is inserted into the inner membrane (Figure 5a,b). The inner face of the cytoplasmic domain has a large negatively charged surface, which probably hinders the collapse of the cytoplasmic domain onto the negatively charged inner cell membrane. Two linkers connecting the two hydrophobic helices to the globular domain are long enough (>7 amino acid residues)—and, thus, modeled with less confidence—which could allow a high degree of structural dynamism for the globular cytoplasmic domain in the range of rotations and distances relative to the transmembrane helices. Such flexibility would allow molecular interactions and the formation of higher‐order macromolecular conformation of the cytoplasmic domain.

Dormancy necessitates the phenomenon of resuscitation back to normal growth conditions; therefore, the state of low metabolic activity has been an appropriate definition to describe dormancy. Remarkably, all differentially upregulated proteins in our earlier studies are central to both metabolic and ribosomal regulatory events for the VBNC state (Mali et al., 2017), which suggests that the Hyp730 family of proteins could play a unique role in dormancy metabolism. A global protein BLAST search shows that Hyp730 and its homologs are highly conserved in only prokaryotes with no redundancy—only one Hyp730‐like gene exists per genome, yet the function of Hyp730 and its homologs are currently unknown. Recently, two cryo‐EM structures of the respiratory alternative complex III (ACIII) with cytochrome oxidase function from *Flavobacterium johnsoniae* (Sun et al., 2018) and *Rhodothermus marinus* (Sousa et al., 2018) were determined and contain an ActD domain, which adopts a similar 3D‐structure to the Hyp730 model and seems to be present in all ACIIIs. In these bacteria, ACIIIs function as membrane‐bound proton pumps that catalyze an elaborate bifurcated electron transfer, coupled to an energy‐saving two‐ or three‐proton translocation in the opposite direction—notably more efficient than the cytochrome bc_1_/b_6_f complexes (Refojo et al., 2012). Accordingly, one possibility is that Hyp730 could act as a sequestering unit of multi‐domain respiratory complexes that are upregulated and indeed require sequestration for effective energy production during dormancy, and ultimately helps produce and conserve energy more efficiently.

In a greater perspective, differentially expressed genes and proteins in our proteomics and transcriptomics mostly belong to cytoplasmic molecules of *M*.* luteus*, suggesting that the cell cytoplasm is the major location where dormancy is programmed. Therefore, dormancy‐specific Hyp730 is unlikely to play a role in the mechanism by which bacteria move to the dormant phenotype, but instead likely facilitates dormant bacteria resuscitation. Rv1234, the Mtb paralog of Hyp730, has previously been labeled as a non‐essential gene for bacterial growth; yet, the essentiality of these genes in dormancy has not been tested. Further direct and conditional knock‐out studies under different stress conditions (such as hypoxia, nutrient deprivation) are ongoing and vitally needed to investigate the physiological role(s) of Hyp730 in different growth stages and further design small molecules that could target Hyp730 function(s).

## CONFLICT OF INTEREST

None declared.

## AUTHOR CONTRIBUTION

**Mehmet Sen:** Conceptualization (lead); Data curation (supporting); Funding acquisition (lead); Methodology (lead); Project administration (lead); Software (lead); Supervision (lead); Writing‐original draft (lead); Writing‐review & editing (lead). **Steven J. Bark:** Methodology (supporting); Resources (supporting). **William R. Widger:** Investigation (supporting); Methodology (supporting); Writing‐original draft (supporting). **Preethi Gunaratne:** Methodology (supporting); Resources (supporting). **Jerry O. Ebalunode:** Conceptualization (equal); Data curation (equal); Methodology (equal); Software (equal); Validation (equal); Visualization (equal); Writing‐original draft (equal); Writing‐review & editing (equal). **Arshad Khan:** Methodology (supporting); Writing‐original draft (supporting). **Sujina Mali:** Methodology (equal); Resources (supporting). **Brandon Mistretta:** Data curation (equal); Software (equal). **Tannon Yu:** Methodology (equal); Writing‐original draft (equal); Writing‐review & editing (equal). **Abiodun P. Bodurin:** Data curation (equal); Formal analysis (equal); Methodology (equal); Software (equal); Writing‐original draft (supporting). **Jonathan Rangel:** Conceptualization (equal); Methodology (equal); Writing‐original draft (equal); Writing‐review & editing (supporting). **Stewart Fannin:** Conceptualization (supporting); Data curation (equal); Formal analysis (equal); Investigation (equal); Methodology (equal); Writing‐original draft (equal); Writing‐review & editing (equal).

## ETHICS STATEMENT

None required.

## Data Availability

All data are provided in full in the results section of this paper.
